# Letter from Paris

**Published:** 1856-01

**Authors:** Robert P. Harris

**Affiliations:** Paris


### LETTER FROM PARIS.

* * * * *Paris, Nov. 19th*, 1855.

My knowledge, as yet, of the Parisian Hospitals, is necessarily very superficial, as I have only visited them during the past week. Last Monday, most of the regular hospital clinics commenced, and to-day the first introductory of the course of lectures at the school of Medicine was delivered. During the time above alluded to, I have visited Hôpital St. Louis, famous for the treatment of skin diseases; Hôpital des Enfants Malades, or sick children's Hospital; Hotel Dieu, a large general hospital, perhaps the oldest in Europe; Hôpital Necker, where Civiale, the celebrated inventor of the lithotriteur, lectures, and Hôpital Clinique de la Faculté de Medecine, for surgical cases and midwifery.

In the commencement of a course of clinical attendance in a foreign country, no matter how well one may understand the language of that country in a general sense, one necessarily loses a great deal of what is said by the lecturer, in consequence of the peculiar form of idiomatic and technical language employed by him. Although I can read French pretty well, and speak it so as to answer all the purposes in which I find it necessary in ordinary daily life, still, when I listèn to a lecture I find very great difficulty in following the person who speaks, so as to understand his entire meaning. This is of course only to be overcome by time and close attention. If the words pronounced by the speaker were given me in a book, I could read them without any difficulty; but as they come from the mouth of a Frenchman, they are so much blended together and curtailed of their syllabic sounds that one cannot tell where one word ends and another begins. This is to me, now, one of the greatest difficulties in the French language. The opportunities of studying French in Paris are much more limited than for acquiring a knowledge of English with us, or in England. A French boarding house is anything but a family residence. Every one in it, is totally independent of every other person, and one may live in a house for months without knowing the name of his next door neighbor, if he so wishes. There is no family table where all talk over the news of the day, and no family fireside where, as in our boarding houses, all the boarders may meet together of an evening, and where a foreigner may every day have a fine opportunity of improving himself in the language of the country. If one wants to talk, he must go and visit his friends, and as these are almost necessarily those who speak his own language, there is little progress made in the French. Most persons who come to Paris and desire to learn the language take French lessons, which is very little more than can be done at home. They talk to the French teacher for an hour a day, and flatter themselves that they are making rapid progress, because he or she, as it may be, tells them so, as a matter of politeness. The next thing is to try their powers of conversation with the concierge or door keeper, who being generally an uneducated person, speaks a patois not desirable to be learned by the pupil. After this, if the student is a medical man, he buys some medical books and reads them, and trys to hear and understand the lectures at the hospitals and school of medicine. About the time he is to go home, he begins to understand what the lecturers say and to profit by their teaching ; but his money is all gone, and he cannot stay any longer, so buying a box of the newest medical publications and some instruments he takes passage for home, and says very little about this the dark side of the picture.

This is the history of many of the students who come here from other countries : but there are exceptions. One who comes here with a good knowledge of French may learn much, particularly if he has been in the habit of reading French medical books. His only difficulty at first, is the catching of the sounds as they come from the mouth of the speaker, but this he will soon overcome. Bedside instruction in Paris is the most profitable and fortunately the most easily understood by the student. The lectures are very little attended by graduates in medicine, unless it be those delivered in the amphitheatres of the hospitals, which are generally followed by surgical operations.

The hospitals of Paris are very clean, and though not very well ventilated are much better than some of our institutions at home in this respect. The best heated and ventilated hospital is said to be the Lariboisiére, erected last year. I have not yet seen it, but hope to pay it a visit in a few days. It is very far from where I live. The hospitals of Paris are all connected with one general office, which supplies funds, &c., and to which all applications are made for admittance, &c. &c. The different wards of the hospitals are named after the various favorite Roman Catholic saints, and the nurses or chiefs of the wards are Sisters of Charity, who I must say are the best I have ever seen. Being generally well educated and intelligent, and not being at all influenced by pecuniary motives, they carry out the designs of the institutions committed to their care with a good will that money will not procure with us. The architecture of the Parisian hospitals is not in the least ornamental, except in one or two instances; in fact the buildings present in many respects rather an unsightly appearance from the street side. They generally have quite extensive grounds connected with them, and some of them are built in the form of a hollow square, having a garden in the centre and all around the building, except on the side next to the entrance. The most extensive grounds and the best kept of any of the institutions I have yet seen, belong to the children's hospital. This hospital is to me the most interesting in Paris. It contains 600 beds, and provision is made for both surgical and medical patients. In the yard is a fine gymnasium, and at present they are building a handsome stone chapel to take the place of one in the main hospital, which will be then changed into a ward for patients. The physicians and surgeon visit the little ones every morning at eight o'clock, and on Thursday Dr. Guersant lectures in the amphitheatre at nine, and performs such operations as may be found necessary. He is a man of medium height, rather stout, of pleasant manners, and a good lecturer. He operated last week for double hare-lip on an infant twelve days old, and upon a little boy of five or six for prolapsus ani. The latter by the actual cautery. At present there are in the house a number of children suffering from scrofula in its various forms, as there will always be in children's hospitals. Some four or five amputations were doing very well, which had been performed for disease of the knee joint. There were several cases of coxalgia, but most of them were in the suppurative stage of the disease. In the medical wards there are at present many cases of disease resulting from cold; such as croup, pneumonia, pleuritis, bronchitis, &c. &c. One little fellow had been operated on by tracheotomy and appeared to be doing very well. Chloroform is used in the operations of this establishment, and is employed also when, in disease of the eye, an application cannot be properly made, or a sight of the inflamed ball obtained under ordinary examination.

The Hotel Dieu, being a general hospital and having nearly nine hundred beds, is very much visited by medical students connected with the school of medicine. It is not as good a school as our hospital, as the instruction given is much more superficial. As the physicians go to the hospitals every day, they do not say much about a patient except the first time of visiting him; and as the examinations are made in the consultation room before the patient enters, the students have but little opportunity of studying what is to them very important, the commencing examination and treatment of the cases. In the diagnosis of the symptoms which present themselves in the course of disease, they have a good chance of gaining instruction, but in the treatment, we are much better physicians than the French, and have as a general rule little to learn from them which would not be better learned at home. In diagnosis they are ahead of the rest of the world, and perhaps also in dietetics. They are capital theorists, but everything must give way to theory when it comes to practice, and in this way they fail of becoming good practitioners. They establish first a theory, and then adapt the practice to this theory, without allowing the practice to prove the truth or falsity of the theory.

In the Hôpital Clinique, there are a number of cases of midwifery under the care of Dr. Dubois, accoucheur to the Empress. He is an able physician, and his bedside lectures are valuable. I have seen him but once, and remarked his extreme closeness of attention to any little matters which might indicate diseased tendency or action in his patients. Very few of his cases had anything remarkable about them. One was threatened with metritis, though the symptoms were not well developed. This is the only obstetric hospital where male students can attend in Paris. In Vienna the school is very superior in this respect, as it is also in Dublin. In the former they have a hospital of eight thousand cases per annum, and in the latter of two thousand.

Yours, Robert P. Harris, M. D.

